# 870. Olorofim for the treatment of invasive mould infections in patients with limited or no treatment options: Comparison of interim results from a Phase 2B open-label study with outcomes in historical control populations (NCT03583164, FORMULA-OLS, Study 32)

**DOI:** 10.1093/ofid/ofac492.063

**Published:** 2022-12-15

**Authors:** Johan A Maertens, Paul E Verweij, Evan F Lanuza, Emma L Harvey, Aaron Dane, Daniela Zinzi, John H Rex, Sharon C Chen

**Affiliations:** KU Leuven, Leuven, Brussels Hoofdstedelijk Gewest, Belgium; Radboud University Medical Center, Nijmegen, Gelderland, Netherlands; Kern Medical, Paso Robles, California; F2G Ltd, Egham, England, United Kingdom; Danestat Consulting Limited, Macclesfield, England, United Kingdom; F2G, Manchester, England, United Kingdom; F2G, Limited, WELLESLEY HILLS, MA; Westmead Hospital, Sydney, New South Wales, Australia

## Abstract

**Background:**

Olorofim is a novel antifungal agent active against *Aspergillus* spp (including azole-resistant strains), rare, resistant moulds (e.g., *Lomentospora prolificans)* and dimorphic moulds.

Serial images of Lomentospora prolificans infection following breast enhancement surgery: progression of healing pre- and post-olorofim therapy.

Post-surgical bone/ soft tissue Lomentospora prolificans infection of the chest wall in a healthy woman was uncontrolled with available agents (D -9 visible mould in wound base). At D42/84 overall response on olorofim monotherapy was stable; wound closure with complete resolution of IFI was achieved at D322 (case previously reported, ECCMID 2020 abstract #2585).

**Methods:**

Patients with limited/no treatment options for proven invasive fungal infection (IFI) or probable pulmonary invasive aspergillosis (IA) using EORTC-MSGERC criteria^1^ received oral olorofim (150mg BID x1d loading dose then 90mg BID). Outcomes in the first 100 patients are compared with historical controls (HCs) as well as with expected outcomes in patients with baseline highly active, uncontrolled IFI (HAU-IFI).

**Results:**

All-cause mortality in IA at month 3 (includes data to Day 100, the best-fit time point for IA HC data) was 17/53 (32%, 95 CI 20–46%) for olorofim vs. 40/46 (87%, 74–95%) in HCs given either no therapy or azole monotherapy for azole-resistant IA. Successful EORTC-MSGERC overall response^2^ (OR, complete or partial based on clinical + radiologic + mycologic improvement) was 47%/42% in IA (Day 42/D84, n = 53), 53%/53% (*L. prolificans*, n=17), 55%/36% (*Scedosporium,* n=11), and 50%/50% (other moulds, n=8). Stable response at D42/D84 predicted extended therapy responses, especially in HAU-IFI of brain and bone (Figure). For *Coccidioides* (n=11) OR was limited to stable due to very slow clearance of fungal serology but clinical response was rapid. Symptoms resolved completely in 18% (2/11) by D84 vs 3% (1/29) by D1523 in comparable HCs with poorly controlled extrapulmonary *Coccidioides* infection; similar trends were seen for other response measures.

**Conclusion:**

Olorofim is a novel oral antifungal with activity against a wide range of mould infections which are difficult to treat. Compared with relevant HCs or expected outcomes for HAU-IFI, olorofim has a positive benefit-risk profile in a well-defined population of patients with limited/no treatment options. As noted previously^3^, considering stable in overall success if often appropriate when assessing responses in non-IA mould IFI.

References:

1. Donnelly CID 2020; 71:1367–76

2. Segal CID 2008; 47:674–83

3. Perfect Mycoses 2018: 61:420

**Disclosures:**

**Johan A. Maertens, MD PhD**, F2G Ltd: Advisor/Consultant|Gilead Sciences Ltd: Advisor/Consultant|Mundipharma: Advisor/Consultant|Pfizer Inc: Advisor/Consultant **Paul E. Verweij, PhD**, Gilead: Grant/Research Support **Emma L. Harvey, MBBS**, F2G Ltd: Stocks/Bonds **Aaron Dane, MSc**, Amplyx: Advisor/Consultant|AN2 therapeutics: Advisor/Consultant|Artizan: Advisor/Consultant|Cidara: Advisor/Consultant|ContraFect: Advisor/Consultant|Correvio: Advisor/Consultant|Davolterra: Advisor/Consultant|Destiny Pharma: Advisor/Consultant|Entasis: Advisor/Consultant|F2G Limited: Advisor/Consultant|GSK: Advisor/Consultant|Humanigen: Advisor/Consultant|Kymab: Advisor/Consultant|Modis: Advisor/Consultant|Orca: Advisor/Consultant|Pfizer: Advisor/Consultant|Phico: Advisor/Consultant|Pled Pharma: Advisor/Consultant|Rare Thyroid: Advisor/Consultant|Roche: Advisor/Consultant|Scynexis: Advisor/Consultant|Sinovent: Advisor/Consultant|Spero Therapeutics: Advisor/Consultant|Transcrip: Advisor/Consultant|Venatorx: Advisor/Consultant **Daniela Zinzi, Infectious Diseases Specialist**, F2G: F2G employee|F2G: Stocks/Bonds **John H. Rex, MD**, Advent Life Sciences: Operating Partner|Advent Life Sciences: Ownership Interest|AMR Action Fund: Advisor/Consultant|AstraZeneca: Stocks/Bonds|Basilea Pharmaceutica: Advisor/Consultant|Bugworks Research, Inc.: Advisor/Consultant|F2G, Limited: Employee|F2G, Limited: Stocks/Bonds|Forge Therapeutics: Advisor/Consultant|GlaxoSmithKline: Advisor/Consultant|Pfizer Pharmaceuticals: Honoraria|Sumitovant: Advisor/Consultant **Sharon C. Chen, PhD MBBS**, F2G PTy Ltd: Grant/Research Support|MSD Australia: Grant/Research Support.

